# Special Issue: “New Challenges of Parkinson’s Disease”

**DOI:** 10.3390/ijms26199564

**Published:** 2025-09-30

**Authors:** Cristina Agliardi, Franca Rosa Guerini

**Affiliations:** IRCCS Fondazione Don Carlo Gnocchi ONLUS, via Capecelatro 66, 20148 Milan, Italy; fguerini@dongnocchi.it

Parkinson’s disease (PD) is the second most common neurodegenerative disorder affecting more than 10 million people worldwide [1]. It is marked by progressive dopaminergic neuronal loss in the *Substantia nigra* and pathological misfolded α-synuclein aggregation that leads to typical motor symptoms (tremors, rigidity, bradykinesia, postural instability) [2]. While dopamine depletion is closely linked to motor deficits, the emergence of certain non-motor symptoms, such as depression, behavior change, psychosis, hallucinations (in particular visual), is complex and likely multifactorial [1]. For instance, visual hallucinations and psychosis can arise not only from the underlying pathology, but also as side effects of long-term dopaminergic treatment, particularly with L-DOPA [3,4]. Moreover, cognitive decline and dementia are common in advanced PD and are strongly associated with widespread α-synuclein pathology beyond the dopaminergic system [5]. As a consequence, all of these symptoms strongly impact the patients’ quality of life.

Early and accurate diagnosis is crucial not only for improving prognostic outcomes, but also for enabling timely therapeutic interventions. However, early diagnosis remains challenging, as the hallmark symptoms of PD often overlap with those of other neurodegenerative diseases [6]. Moreover, current diagnostic practices primarily rely on clinical assessments [7], highlighting the need for more refined and objective diagnostic tools. In this context, the identification and validation of reliable biomarkers are of growing importance, as they can support differential diagnosis, monitor disease progression, and guide the development of personalized treatment strategies, ultimately improving patient care and quality of life [8,9,10].

This Special Issue of the *International Journal of Molecular Sciences* features four reviews and six original research articles that explore new challenges in understanding, diagnosing, and treating PD.

The contributions in this issue cover a broad spectrum of topics, ranging from the identification of biochemical, genetic, and epigenetic biomarkers to the application of advanced approaches such as multi-omics integration, artificial intelligence, and functional imaging. Particular attention is also given to the innovative use of extracellular vesicles (EVs) and to emerging therapeutic strategies. The review articles provide an overview of several recent developments. Arya et al. in Contribution 1 categorize PD biomarkers into biochemical, genetic, and neuroimaging domains, highlighting the diagnostic and prognostic potential of α-synuclein assays, oxidative stress markers, and mitochondrial-derived peptides. Notably, the α-synuclein Seed Amplification Assay (α-synuclein-SAA) represents a paradigm shift, enabling the detection of misfolded α-synuclein aggregates with high sensitivity and specificity. Advances in genetic studies, including GWAS and artificial intelligence (AI)-based approaches, have identified numerous PD-associated loci and revealed key pathogenic mechanisms such as lysosomal dysfunction and impaired stress responses. Emerging evidence also underscores the role of epigenetic regulation, particularly miRNAs, which show strong discriminative power and potential for personalized therapeutic strategies.

Hwang et al. in Contribution 2 demonstrate that multi-omic integration, particularly combining proteomic and miRNome data, can uncover key pathogenic mechanisms in PD including oxidative stress, mitochondrial dysfunction, and impaired neurotransmission. Artificial intelligence enhances this approach by extracting predictive patterns from complex datasets, thereby facilitating the discovery of novel therapeutic targets. Complementarily, Gualerzi et al. in Contribution 3 highlight the diagnostic and therapeutic potential of EVs, which carry disease-relevant proteins and miRNAs, enable differential diagnosis, and correlate strongly with clinical severity.

Klokkaris and Migdalska-Richards in Contribution 4, together with Gualerzi et al. in Contribution 3, review emerging therapeutic strategies for PD, focusing on epigenetic interventions and EV-based drug delivery. Epigenetic approaches—including HDAC inhibition, RNA interference, CRISPR/Cas9 editing, and miRNA modulation—show promise in reducing α-synuclein pathology and improving neuronal viability, although their efficacy requires careful modulation to avoid off-target effects. EVs, with their ability to cross the blood–brain barrier and deliver therapeutic molecules such as siRNA, dopamine, or anti-inflammatory compounds, represent a highly innovative but still experimental platform facing challenges in large-scale production and standardization.

The original research articles of the present issue are based on both animal models and clinical experimental research; they illustrate the diversity of mechanisms implicated in PD pathogenesis and highlight novel therapeutic strategies ranging from microbiota modulation to immune cell therapy.

The pathogenic role of α-synuclein remains a cornerstone of PD research. Barnes et al. in Contribution 5 demonstrated that both soluble and insoluble α-synuclein fractions from a transgenic mouse model induce pathology independent of injection site, suggesting that neuronal vulnerability rather than the site of entry dictates disease progression. Complementing this, Seo et al. in Contribution 6 reported that decreased serum/glucocorticoid-related kinase 1 expression in the intestine correlates with increased α-synuclein levels, providing evidence of gut–brain axis involvement in PD pathology. Gastrointestinal disorders are some of the major prodromal symptoms of PD. Together, these studies underscore the systemic dimension of α-synuclein aggregation and propagation.

Mounting evidence highlights the gut microbiota as a modulator of levodopa pharmacokinetics. Ai et al. in Contribution 7 revealed that targeted microbial modulation enhances levodopa bioavailability and motor recovery in PD models, suggesting that microbial composition explains interindividual therapeutic heterogeneity. Parallel to microbial factors, intracellular peptides may represent another underexplored contributor. Fiametti et al. in Contribution 8 identified five peptides that ameliorated motor deficits in a zebrafish PD model without cytotoxicity, pointing to peptides as potential therapeutic agents with neuroprotective roles.

Additionally, inflammation and mitochondrial dysfunction have long been suspected and explored in PD. Gevezova et al. in Contribution 9 provided evidence that YKL-40, an inflammatory glycoprotein, correlates with altered mitochondrial bioenergetics in PD patients. Elevated YKL-40 levels were associated with increased respiratory reserve and ATP production, suggesting that YKL-40 could serve as both a biomarker and a mechanistic link between neuroinflammation and bioenergetic dysfunction.

An innovative immunological strategy was proposed by Park et al. in Contribution 10, who developed α-synuclein-specific regulatory T cells (Tregs). These cells ameliorated PD progression in mice by reducing motor deficits, dopaminergic neuronal loss, and microglial activation. This approach illustrates how harnessing adaptive immunity could provide disease-modifying effects beyond symptomatic treatment.

Collectively, these studies exemplify the multifactorial nature of PD pathogenesis and therapeutic development. α-Synuclein remains central, but the microbiota, peptides, metabolic regulators, and immune responses are increasingly recognized as integral players. Future research should focus on integrating these domains to design combination therapies, possibly involving microbiota-targeted interventions, peptide-based neuroprotective agents, and immune modulation. Moreover, novel biomarkers may enable patient stratification for precision medicine approaches for both pharmacological and rehabilitative therapies.

The reviewed studies reinforce PD as a systemic disorder involving complex cross-talk between neuronal, immune, microbial, and metabolic networks. Advancing from bench to bedside will require multidisciplinary strategies that target not only the central nervous system, but also peripheral contributors such as the gut and immune system. Together, these findings herald a new era of integrative therapeutics for PD.
